# A Novel Description of the Human Sinus Archaeome During Health and Chronic Rhinosinusitis

**DOI:** 10.3389/fcimb.2020.00398

**Published:** 2020-08-06

**Authors:** Brett Wagner Mackenzie, Annie G. West, David W. Waite, Christian A. Lux, Richard G. Douglas, Michael W. Taylor, Kristi Biswas

**Affiliations:** ^1^Department of Surgery, The University of Auckland, Auckland, New Zealand; ^2^School of Biological Sciences, The University of Auckland, Auckland, New Zealand

**Keywords:** human microbiome, archaea, bacteria, 16S rRNA gene, chronic rhinosinusitis, Droplet Digital™ PCR

## Abstract

Human microbiome studies remain focused on bacteria, as they comprise the dominant component of the microbiota. Recent advances in sequencing technology and optimization of amplicon sequencing protocols have allowed the description of other members of the microbiome, including eukaryotes (fungi) and, most recently, archaea. There are no known human-associated archaeal pathogens. Their diversity and contribution to health and chronic respiratory diseases, such as chronic rhinosinusitis (CRS), are unknown. Patients with CRS suffer from long-term sinus infections, and while the microbiota is hypothesized to play a role in its pathogenesis, the exact mechanism is poorly understood. In this cross-sectional study, we applied a recently optimized protocol to describe the prevalence, diversity and abundance of archaea in swab samples from the middle meatus of 60 individuals with and without CRS. A nested PCR approach was used to amplify the archaeal 16S rRNA gene for sequencing, and bacterial and archaeal load (also based on 16S rRNA genes) were estimated using Droplet Digital™ PCR (ddPCR). A total of 16 archaeal amplicon sequence variants (ASVs) from the phyla *Euryarchaeota* and *Thaumarchaeota* were identified. Archaeal ASVs were detected in 7/60 individuals, independent of disease state, whereas bacterial ASVs were detected in 60/60. Bacteria were also significantly more abundant than archaea. The ddPCR method was more sensitive than amplicon sequencing at detecting archaeal DNA in samples. Phylogenetic trees were constructed to visualize the evolutionary relationships between archaeal ASVs, isolates and clones. ASVs were placed into phylogenetic clades containing an apparent paucity of human-associated reference sequences, revealing how little studied the human archaeome is. This is the largest study to date to examine the human respiratory-associated archaeome, and provides the first insights into the prevalence, diversity and abundance of archaea in the human sinuses.

## Introduction

Human-associated microbial communities are diverse and occupy site-specific niches. These microbes are intrinsically linked to human health, by helping maintain homeostatic functions and contributing to both acute and chronic disease. To date, most human microbiome research has focused on bacteria, as they are the dominant members of the microbiome (Sender et al., 2016) However, recent advances in sequencing technologies have expanded research to include fungi, other eukaryotes, archaea, and viruses (Hoffmann et al., 2013; Lim et al., 2015; Monaco et al., 2016; Hannigan et al., 2018; Hoggard et al., 2019). The composition and contribution of these lesser-known members to both health and disease states remains an exciting area for human microbiome research.

Species of the domain Archaea are characterized by a unique cell wall structure that assists survival in extreme conditions such as hydrothermal vents, salt lakes, anoxic and highly acidic or alkaline environments (Eichler, 2003). Recent research suggests that archaea may be as widely distributed as bacteria and colonize a diverse range of hosts (Lloyd et al., 2013; Moissl-Eichinger et al., 2018). Most of the human microbiome research investigating the archaeome (the archaeal portion of the human microbiome) focuses on the gut, where methanogens are the dominant archaea (Miller and Wolin, 1985; Dridi et al., 2009; Miragoli et al., 2017; Wampach et al., 2017). More recently, archaeal signatures have been detected in subgingival dental plaque, skin, lung, sinus and nares samples (Lepp et al., 2004; Dridi et al., 2011; Hulcr et al., 2012; Probst et al., 2013; Oh et al., 2014; Koskinen et al., 2017; Moissl-Eichinger et al., 2017; Pausan et al., 2018; Wagner Mackenzie et al., 2018).

The lack of any known archaeal pathogen presumably contributes to the paucity of studies investigating the prevalence, composition and role of the human archaeome, especially during health and disease. No studies to date have specifically examined the composition or diversity of the sinus archaeome. Of particular interest is the composition of the sinus microbiome during health and chronic rhinosinusitis (CRS). CRS is a disease affecting up to 6.4% of the population and characterized by long-term inflammation of the sinuses (Dietz de Loos et al., 2019). CRS is associated with both a substantial impact on the quality of life of patients that suffer from this disease and high health care costs (Rudmik, 2017; Bhattacharyya et al., 2019). Although the microbiome is hypothesized to play a role in the etiology of CRS and/or the exacerbation of symptoms, no microbial pathogen or disease-specific microbial community profile has been identified to date (Hoggard et al., 2017; Cho et al., 2020). The overwhelming proportion of human DNA from sinus samples has so far limited the insights gleaned from metagenomic studies, therefore many studies employ amplicon sequencing techniques to focus on bacterial associations with disease. The only study to date which applied metagenomic sequencing to CRS sinus samples reported the presence of archaea, but the absence of healthy subjects and technical controls limited the conclusions that could be drawn (Wagner Mackenzie et al., 2018).

Recent studies have evaluated primer-pairs and data processing pipelines for targeting the archaeal component of the human microbiome for amplicon sequencing (Koskinen et al., 2017; Pausan et al., 2018). Studies by Pausan et al. optimized the detection and quantification of archaea in a variety of samples from different sites, including swab samples from the upper nasal cavity. Although the total number of samples investigating the upper respiratory archaeome was small [Koskinen et al. (*n* = 2) and Pausan et al. (*n* = 7)] a diverse range of archaea were nonetheless detected.

In this cross-sectional study, we applied the optimized protocols by Pausan *et al*. to investigate the prevalence, diversity and abundance of archaea in the human sinuses and any associations with disease state. Specifically, we used 16S rRNA gene amplicon sequencing to assess microbiota composition, and Droplet Digital™ PCR to estimate total bacterial and archaeal loads.

## Materials and Methods

### Sample Collection

For this study, 40 participants undergoing endoscopic sinus surgery and 20 healthy volunteers with no history of sinus disease, asthma, or recent antibiotic usage (≤6 months) were recruited. Of the 40 that underwent surgery, participants had CRS with nasal polyps (CRSwNP) (*n* = 16), CRS without polyps (CRSsNP) (*n* = 15), or were disease control participants (*n* = 9). Disease control participants were defined as patients undergoing endoscopic sinonasal surgery for reasons unrelated to CRS. The bacterial composition of the 40 participants undergoing surgery has been published previously as part of a different study (Wagner Mackenzie et al., 2019).

All participants were ≥18 years of age. CRS patients with immunodeficiencies or vasculitis were excluded. Pairs of sterile, endoscope-guided rayon swab samples (Copan Diagnostics, Inc., Murrieta, CA) were collected from the left middle meatus of all individuals. Swab samples were stored in RNA*later*™ (Thermo Fisher Scientific, New Zealand) at 4°C overnight, then transferred to −20°C until processed. This study was approved by the New Zealand Health and Disability Ethics Committee (NTX/08/12/126) and all patients provided informed consent.

### Nucleic Acids Extraction and Target Gene Amplification

Samples from healthy volunteers were thawed on ice, and DNA was extracted from pairs of swabs using the Qiagen® AllPrep DNA/RNA Mini Kit (Bio-Strategy Ltd, Auckland, New Zealand) as previously described (Wagner Mackenzie et al., 2019). DNA from all samples, including those from the previous study, was extracted in a consistent manner. Briefly, after bead-beating to lyse cells and two wash steps to remove impurities, sterile PCR-grade water (32 μL) was added to the spin column filter and incubated for 5 min before DNA was eluted by centrifuging for 1 min at 11,200 × g. The eluate was centrifuged through the spin column filter a second time to increase DNA concentration. A negative extraction containing 200 μL of sterile water and PCR amplification of PCR-grade water was performed to assess for microbial contamination of the DNA extraction kit and PCR reagents.

A nested PCR approach using primers optimized by Pausan et al. was applied to amplify the bacterial and archaeal 16S rRNA gene (Pausan et al., 2018). Briefly, up to 100 ng of template DNA from each of the 60 participants was amplified in duplicate. For the first PCR, each reaction was performed in a final volume of 20 μL that included: 2.5 μL of 10X Buffer, 10 mM MgCl_2_, 2.5 mM dNTPs, 0.1 μL HotStar DNA Polymerase (Qiagen), and 0.5 μL of 10 mM of each primer S-D-ARCH-0344-a-S-20 (ACG GGG YGC AGC AGG CGC GA) and S-D-ARCH-1041-a-A-18 (GGC CAT GCA CCW CCT CTC), 13.9 μL PCR-grade water, and 1 μL template DNA. Negative controls comprised PCR-grade water, and genomic DNA obtained from *Escherichia coli* (bacterial) and *Halorussus* (archaeal) were used as positive controls in all PCRs. Thermocycling conditions were as follows: 15 min initial denaturation at 95°C, followed by 12 cycles of 94°C for 30 s, 56°C for 45 s, and 72°C for 1 min, with a final extension step at 72°C for 10 min. Amplicons were not purified after the first PCR, to limit the risk of contamination.

Two microliters of the resulting PCR product were transferred into a second PCR containing 2.5 μL of 10X Buffer, 10 mM MgCl_2_, 2.5 mM dNTPs, 0.1 μL HotStar DNA Polymerase (Qiagen), and 0.5 μL of 10 mM of each barcoded primer for Illumina sequencing S-D-Arch-0519-a-S-15 (TCG TCG GCA GCG TCA GAT GTG TAT AAG AGA CAG CAG CMG CCG CGG TAA) and S-D-Arch-0786-a-A-20 (GTC TCG TGG GCT CGG AGA TGT GTA TAA GAG ACA GGG ACT ACV SGG GTA TCT AAT), and 17.9 μL PCR-grade water for a final volume of 25 μL. Thermocycling conditions were as follows: 15 min initial denaturation at 95°C, followed by 35 cycles of 95°C for 30 s, 55°C for 30 s, and 70°C for 40 s, with a final extension step at 70°C for 3 min. Template of controls from the first PCR were included in the second PCR. Additional controls for the second PCR included PCR-grade water, and *E. coli* and archaeal *Halorussus* genomic DNA.

Amplicons from each PCR step were visualized by agarose gel electrophoresis. Replicate, nested PCR products from each sample were purified using Agencourt AMPure beads (Beckman Coulter Life Sciences Inc., USA) according to manufacturer instructions. PCR products were quantified using the High Sensitivity (HS) kit on the Qubit® Fluorometer 1.0 (Invitrogen Co., Carlsbad, USA). Negative DNA extraction and positive *Halorussus* controls were included in purification and sequencing. Purified amplicons were submitted to Auckland Genomics Ltd for library preparation using a dual-indexing approach with Nextera technology and sequencing (2 × 300 bp, paired-end) on Illumina MiSeq. All sequencing data have been deposited with NCBI under BioProject ID number PRJNA599016.

### Bioinformatic Analyses

Amplicon sequence data were processed according to the pipeline recommended in previous human archaeome studies (Koskinen et al., 2017; Pausan et al., 2018) using the open source package DADA2 (Callahan et al., 2016) in R version 3.6.1 RStudio Team (2020) following a modified version of the DADA2 pipeline tutorial v1.12 (https://benjjneb.github.io/dada2/tutorial.html). Briefly, raw sequences were quality filtered using the parameters truncLen=c(240,280) and trimLeft=c(17,21) to remove primers, and maxN=0 and maxEE=c(3,5) to remove poor quality sequences. Quality filtered sequences were merged, denoised, and amplicon sequence variants (ASVs) were inferred. The resulting ASVs were assigned taxonomy using the SILVA v128 database to be consistent with other archaeal sequencing from respiratory-associated samples. Those ASVs that were detected in both the negative DNA extraction control and in samples were either subtracted or completely removed from the final ASV table. It is important to note that 13 sequence counts were detected in the DNA extraction control that were assigned to the same ASV as the positive control (archaeal ASV1, *Halorussus*). This ASV was also detected in the samples, and 13 counts were subtracted from each of the samples where it was detected. No other archaea were detected in the DNA extraction control. Samples were rarefied to an even depth of 11,644 quality-filtered, taxon-assigned ASV sequence counts per sample. The rarefied and contaminant-corrected ASV table was used for all downstream processing unless otherwise noted.

Alpha and beta diversity analyses and taxa plots were generated using “phyloseq” version 1.28.0 package in R version 3.6.1 (McMurdie and Holmes, 2013). Observed ASVs, Shannon, and Inverse Simpson diversity analyses were calculated using the “estimate_richness” command. Box plots were generated using the R package “ggplot2” to visualize alpha diversity results (Wickham, 2016). A Bray-Curtis distance matrix was generated using the “ordinate” function and visualized using the “plot_ordination” command. Permutational analysis of variance was implemented using the “Adonis” command in the vegan package in R version 3.6.1 to assess the contribution of diagnosis to variance in the model (Oksanen et al., 2019).

Differences in microbial communities within groups at ASV level were evaluated. First, ASVs with <0.01% relative abundance across the rarefied dataset were removed. This resulted in 460 taxon-assigned ASVs. Differences between groups were then evaluated using a Kruskal-Wallis rank sum test to generate overall *p*-values that indicated a significant difference existed at least once across the groups, then pairwise comparisons were made between treatment groups using Dunn's test with “BH” *p*-value correction for multiple pairwise comparisons. To visualize differences in abundance of specific ASVs, box plots were generated as previously described.

### Phylogenetic Tree Inference

Phylogenetic trees were constructed to depict the relationship of all the archaeal ASVs identified in this study to known archaeal taxa. Sequences were aligned by using the online SINA alignment tool (version 1.2.11) (Pruesse et al., 2012) which was then imported into ARB version 6.0.6 (Ludwig et al., 2004). For each archaeal phylum (i.e., *Euryarchaeota* and *Thaumarchaeota*), a 50% conservation filter was constructed and used to filter the sequences of ASVs and a manually selected set of reference sequences. Alignments were then exported and maximum-likelihood phylogenetic inference performed with IQ-Tree version 1.6.9 (Nguyen et al., 2015) using the GTR model of nucleotide substitution with Gamma-distributed rate heterogeneity. For each tree, 1,000 bootstrap re-samplings were performed to assess node support. The resulting trees were visualized and color-coded according to the archaeal family level using the Interactive Tree of Life resource version 4 (Letunic and Bork, 2019).

### Quantification of Archaeal and Bacterial 16S rRNA Gene Copies

Droplet Digital™ PCR (ddPCR) was used to measure absolute quantities of bacterial and archaeal DNA in each sample. Droplet generation, PCR amplification, and QX200 droplet readings were conducted using the QX200 Droplet Digital™ PCR System and QuantaSoft™ Software according to the manufacturer's instructions (Bio-Rad Laboratories, New Zealand). Briefly, the V1-V3 regions of the bacterial 16S rRNA gene were amplified using primers 8F and 341R (Biswas et al., 2015). Each ddPCR reaction contained 11 μL EvaGreen®, 0.5 μL 10 μM 8F forward primer, 0.5 μL 10 μM 341R reverse primer, 9 μL of sterile PCR-grade water, and 1 μL of sample DNA for a total volume of 22 μL. A positive control of *E. coli* DNA and a negative control of 1x ddPCR buffer with PCR-grade sterile water were included. Thermocycling conditions were as follows: enzyme activation at 95°C for 5 min, followed by 40 cycles of denaturation at 95°C for 30 s and annealing/extension at 60°C for 1 min. A single signal stabilization step at 4°C for 5 min then 90°C for 5 min was carried out. The same protocol was followed for archaea, substituting bacterial primers with archaeal primers S-D-Arch-0787-a-S-20 (ATT AGA TAC CCS BGT AGT CC) and S-D-Arch-0958-a-A-19 (YCC GGC GTT GAM TCC AAT T) (Pausan et al., 2018). Droplets were analyzed using the QuantaSoft™ Software according to the manufacturer's recommendations. Manual thresholds were set for droplet counts. Box plots were generated to visualize the data as previously described.

### Statistical Analyses

All data were analyzed according to patient diagnosis: healthy, control, CRSsNP, or CRSwNP. All statistical analyses were conducted in R version 3.6.1. Data were checked for normality using the command “normcheck” in the package “s20x” version 3.1-28 followed by ANOVA for normally distributed data or Kruskal-Wallis test for non-normally distributed data. If a significant difference was detected, pairwise comparisons were conducted with Tukey's HSD *post-hoc* test or Mann-Whitney U-test for normal and non-normally distributed data, respectively. *P*-value levels < 0.05 are considered significant unless otherwise stated.

## Results

Demographic statistical analyses revealed significant differences in the age of subjects at the time of sampling between the four cohorts, with healthy subjects being younger (average age ± standard deviation 26.1 ± 7.9 years) than disease control (45.8 ± 6.4), CRSsNP (46.3±13.8), and CRSwNP (52.8 ± 12.5) cohorts (*p* < 0.001). There were significantly fewer females in the CRSwNP cohort (2/16, *p* < 0.05), both CRSsNP and CRSwNP had significantly higher instances of asthma than healthy and disease controls, and CRSwNP patients had significantly higher Lund-Mackay scores than CRSsNP (Table 1).

**Table 1:** Patient demographics and results from statistical analyses.

**Variables**	**Healthy controls (*n* = 20)**	**Disease controls (*n* = 9)**	**CRSsNP (*n* = 15)**	**CRSwNP (*n* = 16)**	**Unadjusted test *p*-value**
Age	26.1 ± 7.9	45.8 ± 6.4	46.3 ± 13.8	52.8 ± 12.5	***p*** **<** **0.001**
European	13/20	6/9	13/15	12/16	*p* > 0.05
Female	9/20	6/9	6/15	2/16	***p*** **<** **0.05**
Lund-Mackay score	NA	NA	13.1 ± 2.8	19.4 ± 4.4	***p*** **<** **0.001**
Asthma	0	0	13/15	6/16	***p*** **<** **0.001**
Antibiotics^*^	1/20	0	0/15	3/16	*p* > 0.05
Never smoked	20/20	7/9	14/15	14/16	*p* > 0.05
Bacterial MiSeq prevalence (100%)	20/20	9/9	15/15	16/16	*p* > 0.05
Bacterial ddPCR prevalence (100%)	20/20	9/9	15/15	16/16	*p* > 0.05
Archaeal MiSeq prevalence (13%)	2/20	1/9	1/15	3/16	*p* > 0.05
Archaeal ddPCR prevalence (85%)	17/20	8/9	14/15	12/16	*p* > 0.05

*Continuous variables were tested for normality using Shapiro-Wilk normality test followed by analysis of variance then Tukey multiple comparisons of means for pairwise comparisons. Means ± standard deviations are shown. Categorical variables were tested using a Fisher's exact test. Significant results (p < 0.05) are shown in bold typeface. MiSeq prevalence refers to detection of ASVs with amplicon sequencing, and ddPCR prevalence refers to Droplet Digital™ PCR detection with Domain-specific primers*.

* *Antibiotic prescription within 4 weeks of sample collection*.

Sequence data quality filtering, correction for contamination, and rarefaction to 11,644 sequences per sample resulted in a total of 2,127 ASVs across 60 samples. The majority of ASVs in the rarefied data table were bacterial (2,111 bacterial ASVs compared to 16 archaeal ASVs). The archaeal positive control was assigned correctly to the archaeal genus *Halorussus*, and no other archaeal ASVs detected in samples were also detected in the positive control. After corrections for contamination, 15 archaeal ASVs were unique to samples.

“Adonis” analyses suggested that diagnosis contributed significantly to differences observed between microbial communities (*R*^2^ = 0.083, *p* = 0.009). The presence of archaea in a subject's microbiome did not contribute significantly to variance observed in the dataset (*p* > 0.05), and presence of archaea in the microbiome was not associated with disease status (*p* > 0.05).

### Archaea Are Less Prevalent, Diverse, and Abundant Than Bacteria

**Figure 1:**
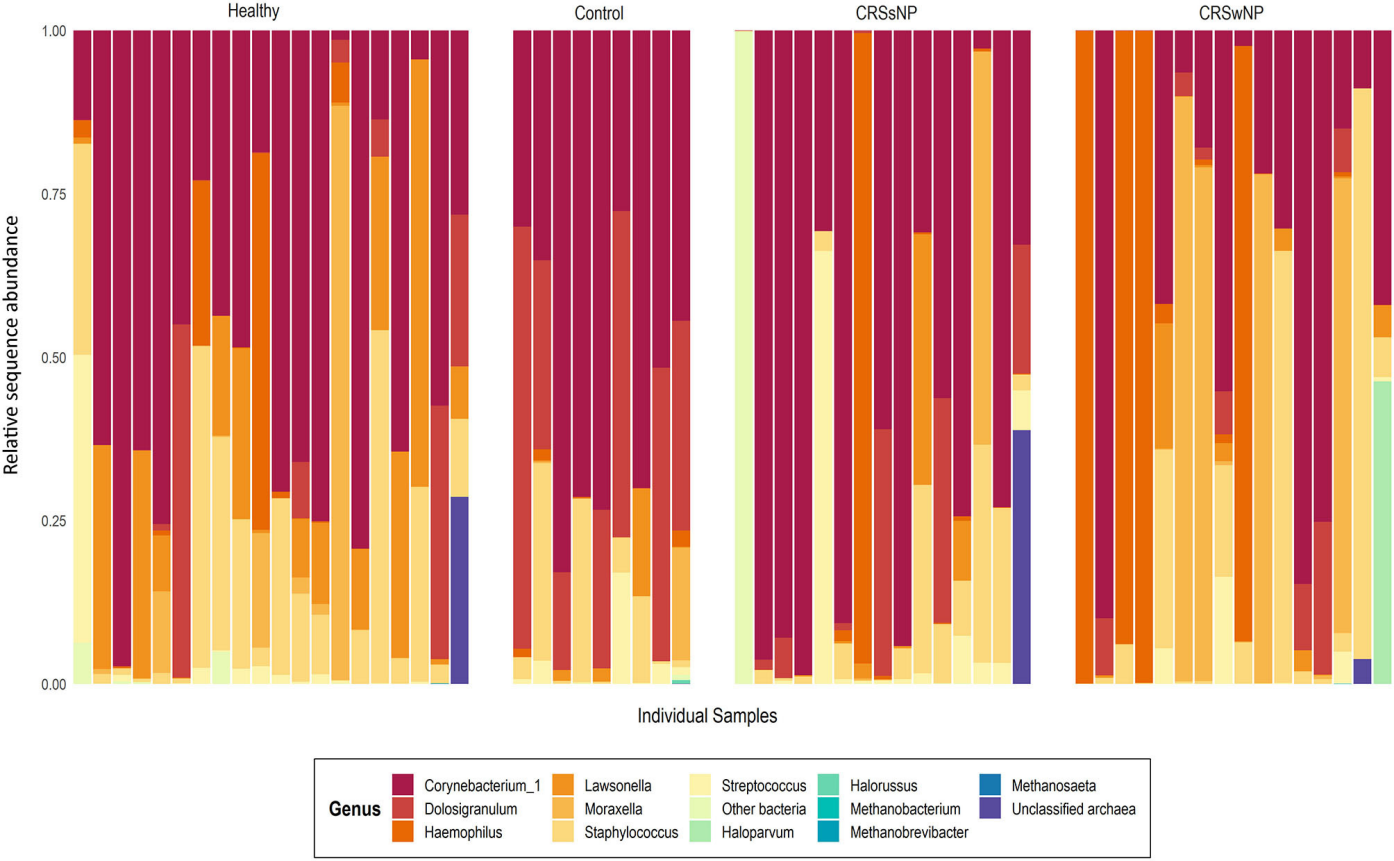
Taxa bar-plot showing the microbial communities recovered from each middle meatus swab sample in this study. Subjects are grouped according to disease state. Relative sequence abundances (%) of the seven most abundant bacterial genera, with all other genera grouped in “Other bacteria,” are shown, as well as all detected archaea classified to genus-level. Other archaeal amplicon sequence variants which could not be classified to genus-level are grouped as “Unclassified archaea”.

Archaeal ASVs were detected in 7/60 subjects (healthy: *n* = 2, disease control: *n* = 1, CRSsNP: *n* = 1, CRSwNP: *n* = 3). Of the 16 ASVs detected across the dataset, 8 were assigned to the archaeal phylum *Euryarchaeota* and 8 to the *Thaumarchaeota* (Table S1). The average relative abundance of archaea in a subject's microbiota, when present (± standard deviation), was 14.7% ± 18.3% (range 0.03–45%). Interpersonal archaeal diversity was high, and no consistent archaeal profile was observed across samples where archaea were detected (Figure 1). Furthermore, in patients where archaeal ASVs were detected, only one or two dominant archaea-assigned ASVs were reported, and these ASVs were members of the same archaeal class. No skewing by disease state according to the archaeal portion of the microbiota was observed.

In contrast to the low prevalence of archaea, bacteria-assigned ASVs were detected in every sample in this study (60/60). The most abundant bacterial ASV, on average, across the entire dataset was assigned to the genus *Corynebacterium_1* (23.3% ± 25.9%). Other prevalent and abundant ASVs included *Staphylococcus, Dolosigranulum, Moraxella, Lawsonella*, and *Haemophilus* (Figure 1). Across the dataset, a total of 23 bacterial phyla were detected. However, the majority of these phyla were detected at very low relative abundances.

**Figure 2:**
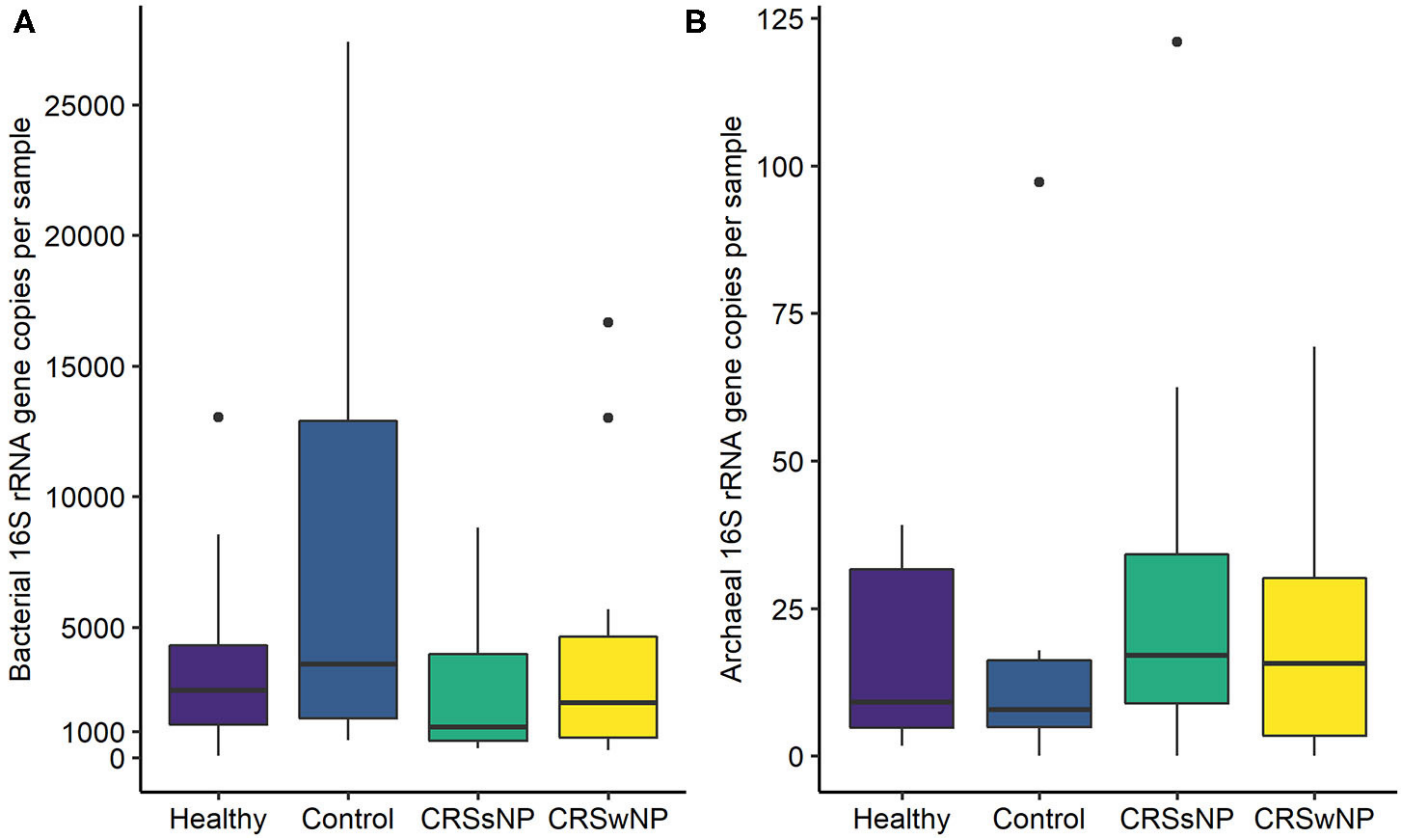
Box plots depicting **(A)** bacterial and **(B)** archaeal 16S rRNA gene copy numbers measured using Droplet Digital™ PCR. Some outlier samples have been removed from graph **(A)** for visualization purposes; outlier datapoints were included in all statistical analyses. No significant differences in bacterial or archaeal loads were observed between groups. Note the differences in y-axes scales between **(A,B)**. Median values are indicated by the solid black line within each box, extending to the upper and lower quartile values.

The absolute quantities of bacteria and archaea in each sample were measured using ddPCR. Bacterial DNA was detected in all samples, with an average number of 23,483 ± 68,862 bacterial 16S rRNA gene copies per sample (Figure 2). ddPCR was more sensitive than Illumina MiSeq amplicon sequencing for detecting archaeal DNA. Archaeal DNA was detected in 51/60 subjects, although at extremely low quantities on average (20 ± 23 archaeal 16S rRNA gene copies per sample). Overall, bacterial load was significantly greater than that for archaea (*p* < 0.0001).

### Archaeal Amplicon Sequencing Reveals Broad Phylogenetic Diversity

Since many of the archaeal ASVs belonging to the phylum *Thaumarchaeota* could not be classified beyond the level of class, phylogenetic trees were inferred to provide greater insight into the archaeal sequences recovered in this dataset. For the phylogenetic tree construction, all archaea-assigned sequences recovered in the dataset before rarefaction were included. Details of the ASV nucleotide sequence, corresponding ASV number used in this study, and taxonomic identification can be found in Table S1. Three additional ASVs recovered from the archaea positive control (ASVs 17, 18, 19—all found at very low counts) were included in phylogenetic reconstructions; these ASVs were not found in any subject samples, but were included nonetheless.

**Figure 3:**
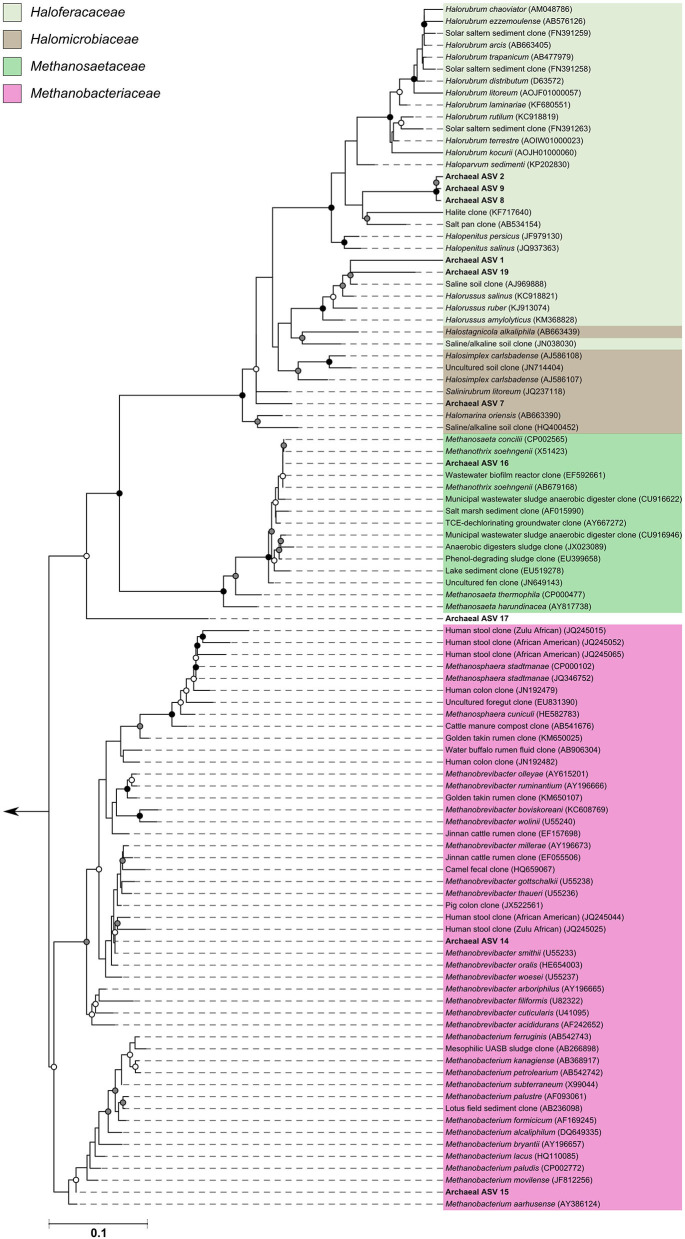
Phylogenetic inference of archaeal amplicon sequences from the middle meatuses of the subjects in this study. Support of internal nodes was determined through 1,000 bootstrap resamplings, and is represented on nodes with >90% bootstrap support (black), 70–90% support (gray), 50–70% support (white). Scale bar represents 1% sequence divergence. The tree is colored according to archaeal families *Haloferacaceae, Halomicrobiaceae, Methanosaetaceae*, and *Methanobacteriaceae*. Corresponding archaeal ASV taxon assignments from this study are found in Table S1.

**Figure 4:**
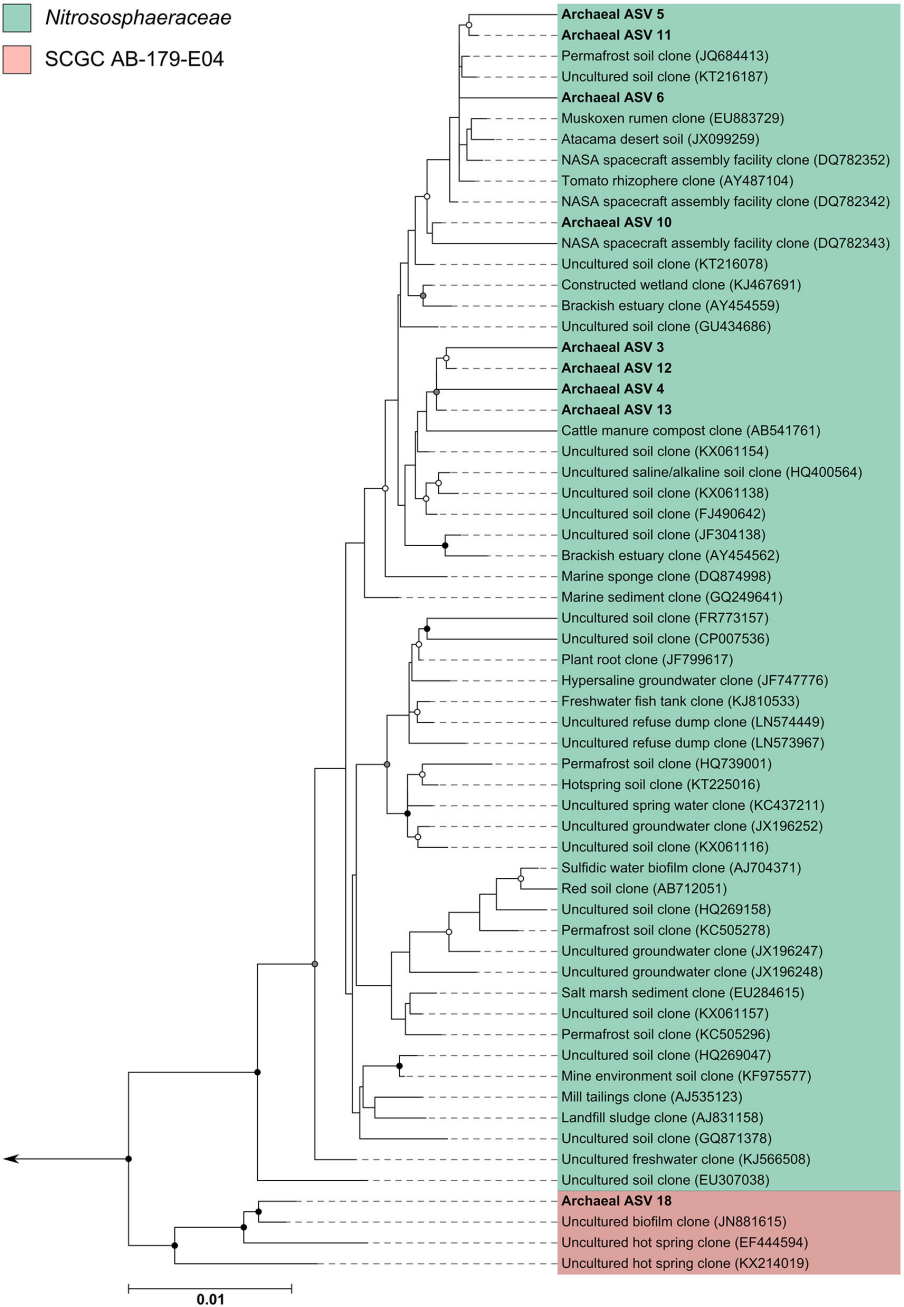
Phylogenetic inference of archaeal amplicon sequences from the middle meatuses of the subjects in this study. Support of internal nodes was determined through 1,000 bootstrap resamplings, and is represented on nodes with >90% bootstrap support (black), 70–90% support (gray), 50–70% support (white). Scale bar represents 1% sequence divergence. The tree is colored according to archaeal families *Nitrososphaeraceae* and *SCGC AB-179-E04*. Corresponding archaeal ASV taxon assignments from this study are found in Table S1.

The archaeal assigned sequences in this study clustered into six archaeal families. Figure 3 depicts the position of archaeal ASVs 1,2,7,8,9,14,15,16,17, and 19 which clustered amongst other archaeal representatives in the *Haloferacaceae, Halomicrobiaceae, Methanosaetaceae*, and *Methanobacteriaceae* families. Archaeal ASVs 3,4,5,6,10,11,12, and 13, which were identified as *Thaumarchaeota*, clustered with isolates belonging to the family *Nitrososphaeraceae* (Figure 4). Archaeal ASV 18, with only 4 sequence counts recovered from the positive control, clustered with the candidate archaeal family *SCGC AB-179-E04*. The representatives of archaea in the inferred phylogenetic trees were isolated from a very diverse range of environments. Congruence of assignments between phylogenetic tree reconstruction and the SILVA v128 sequences was low, highlighting the critical need for advancing both archaeal phylogeny and taxonomy.

### Microbial Characteristics Associated With Diagnosis

While no archaeal ASVs were associated with diagnosis, samples from healthy volunteers had significantly more ASVs on average than those from control, CRSsNP and CRSwNP cohorts (Observed species metric, *p* < 0.001) (Figure S1). Additionally, control participants had significantly higher richness of ASVs when compared with CRSwNP samples. Although healthy volunteers tended to have higher Shannon and Inverse Simpson diversity when compared to other groups this was not significant (Shannon and Inverse Simpson *p* > 0.05).

Pairwise comparisons of ASVs between groups revealed a number of bacteria-assigned ASVs that were significantly differentially abundant (Table S2). No archaeal ASVs were observed as significantly associated with any group. Comparisons between CRSsNP and CRSwNP ASV abundances revealed only one ASV, associated with the bacterial genus *Moraxella*, to be significantly different between the two groups. This ASV was more abundant in CRSwNP (Figure S2). Increased relative abundances of ASV4, associated with *Dolosigranulum*, characterized control subject sinus microbiota. The largest number of significant differences observed between groups was among healthy volunteers compared with controls, CRSsNP and CRSwNP. An increased relative abundance of ASVs from the bacterial genera *Burkholderia-Paraburkholderia, Flectobacillus, Dyella, Ralstonia, Actinomyces*, and *Lawsonella* were associated with healthy volunteers (Figure S2).

Comparisons of bacterial gene copy numbers between groups did not reveal any significant differences (*p* > 0.05). No significant differences were observed in the number of archaeal gene copy numbers between groups (*p* > 0.05).

## Discussion

In this study we applied recently optimized methodologies to evaluate the prevalence, diversity and abundance of archaea in human sinuses during health and chronic disease (Pausan et al., 2018). The diversity and composition of archaea in the sinuses exhibited high interpersonal variation, and we observed no association of archaea with disease state. High interpersonal variation of both archaeal and bacterial communities is a common theme in human microbiome research (Caporaso et al., 2011; Biswas et al., 2015; Hoggard et al., 2016; Lloyd-Price et al., 2017; Pausan et al., 2018), and our results support this observation.

The prevalence and diversity of archaea across the 60 subjects in our study was lower than in other studies; however this may be partly attributed to a number of factors such as sampling method, sequencing depth, sequencing type (amplicon vs. metagenomic) and/or sampling site. In this study, members from both *Euryarchaeota* and *Thaumarchaeota* phyla were detected which have been previously reported. However, we did not recover any members from *Woesearchaeota* which were detected in nares swabs from other human archaeome studies (Koskinen et al., 2017; Pausan et al., 2018). Interestingly, the samples in our study had a very low prevalence and abundance of *Methanobrevibacteriaceae*, which was the main archaeal representative detected in a previous study in our group which applied metagenomic sequencing to sinus swabs (Wagner Mackenzie et al., 2018).

There may be differences in sampling site of the upper respiratory tract (upper nares vs. the sinuses) or geographic differences in the composition of the archaeome. The effect of sampling site within the sinonasal cavity is unclear, and a consensus has not been reached regarding the effect on bacterial community composition (Biswas et al., 2015; Lal et al., 2017; Copeland et al., 2018). Sampling site may play a larger role in the recovery of other less abundant members of the microbiota. Additionally, a recent world-wide study collected middle meatus swabs samples from CRS and control subjects from 13 centers across 5 continents (Paramasivan et al., 2019). Minor, but significant differences in bacterial community composition associated with geographic location were identified. These results suggest that other members of the microbiota, such as archaea and especially fungi, may respond to geographic-driven changes. Together, these effects may partly explain the differences observed between our results and those from other studies.

The human archaeome is drastically understudied when compared with environmental microbiomes. Although the human archaeome is an exciting new area for research, larger studies are required in order to contextualize the results from the few studies published to date. In addition to bacteria and archaea, other microbes such as fungi and viruses are present in the sinuses during health and CRS disease (Lloyd-Price et al., 2017; Zhao et al., 2017; Goggin et al., 2019; Hoggard et al., 2019). Cross-kingdom interactions and co-occurrence patterns in the human airway microbiome should be investigated further. In parallel to amplicon studies that can give insight into archaeal community composition, genome reconstruction of human-associated archaea, either from metagenomic data or directed culturing, should be undertaken in order to ascertain the ecological functions and roles of these organisms. Archaea, in general, are underrepresented in microbial databases, and it is therefore unsurprising that phylogenetic inference clustered the archaeal sequences recovered from the sinus samples in this study with taxa from a diverse array of seemingly unrelated environments. Although we did not observe or identify an association of the archaeal aspect of the sinus microbiota with disease, subsequent studies are necessary as human archaeome research is in its infancy and the archaeal representatives in taxonomic databases are continually updated.

Archaea are characterized by their unique cell wall membrane in addition to differences in DNA repair, genetic features, biochemical and metabolic capabilities (Bang and Schmitz, 2015). These phenotypic and genotypic differences likely result in functionally distinct roles in the human airway microbiome, however, so few studies only allow for speculation. Recent studies suggest that the human immune system recognizes and can be activated by archaea (Hirai et al., 2013; Bang et al., 2014). Furthermore, archaea are characterized by a lack of peptidoglycan in their cell wall, which makes these microbes resistant to a wide spectrum of antibiotics (Khelaifia and Drancourt, 2012). Determining how archaea interact with the host and respond to antimicrobials may help resolve the role of these microbes in both health and disease.

One advantage of employing the primers used in this study is that it allowed us to investigate the bacterial portion of the microbiota in parallel to archaea. The low prevalence of archaea in our study limited our ability to produce reliable correlations between bacteria and archaea. However, a well-established syntrophy exists between methanogenic archaea and bacteria in the gut and this relationship may help explain the presence of methanogens in the sinuses (Samuel et al., 2007). There is also some evidence to support the association of sulfate-reducing bacteria with *M. oralis* in subgingival dental plaque (Nguyen-Hieu et al., 2013). Other studies have hypothesized that low levels of ammonium found on the skin may foster the growth of members from the phylum *Thaumarchaeota* (Probst et al., 2013). The associations of archaea and bacteria in health and chronic diseases may become apparent in subsequent studies with larger sample sizes.

Our results confirmed previous associations of bacterial composition and diversity with CRS disease. Similar to previous studies, we observed significantly increased alpha diversity in the sinus microbiota of healthy subjects when compared with disease control, CRSsNP and CRSwNP subjects (Abreu et al., 2012; Hoggard et al., 2016; Wagner Mackenzie et al., 2016; Cope et al., 2017). It should be noted, however, that the significantly younger age of the healthy subjects compared with disease controls and both CRS cohorts may influence this observation (Aleman and Valenzano, 2019). Interestingly, and in support of a previously published meta-analysis by our group, we observed a significantly increased relative abundance of ASVs assigned as *Burkholderia-Paraburkholderia* and *Ralstonia* with healthy subjects (Wagner Mackenzie et al., 2016). The relative abundances of these ASVs were very low, <1% of total relative abundance on average in healthy subjects for both *Burkholderia-Paraburkholderia* and all three *Ralstonia* ASVs, but were nonetheless significant after corrections for multiple pairwise comparisons. The meta-analysis included published sequences from previous studies, and the samples sequenced here were not included. Members of the *Burkholderiaceae* family (and other skin-associated bacteria) have been associated with DNA extraction kit contamination (Glassing et al., 2016), however in our study we have corrected for contamination. The associations of these taxa with healthy sinuses are intriguing and should be investigated further to ascertain if they are an artifact of methodology or if they are indeed biologically meaningful.

## Conclusion

The results from this study suggest it is unlikely that the archaeal portion of the sinus microbiota significantly influences or is influenced by disease state. However, it is likely that the sinus bacterial community is associated with CRS or is influenced by disease state. The exact nature of this association remains unclear. To our knowledge, this is the largest study to date examining the human associated respiratory archaeome. We observed a lower prevalence, diversity and abundance of archaea when compared with bacteria. We strongly recommend future studies include investigations of archaea in human microbiome samples in order to better understand the role of archaea in human health, but also to better characterize this domain in general.

## Data Availability Statement

The datasets generated for this study can be found in the NCBI under accession number PRJNA599016.

## Ethics Statement

The studies involving human participants were reviewed and approved by the New Zealand Health and Disability Ethics Committee (NTX/08/12/126). The patients/participants provided their written informed consent to participate in this study.

## Author Contributions

BW designed the experiment, conducted lab work, processed sequencing data, interpreted results, and wrote the manuscript. AW and DW contributed to data analyses and manuscript review. CL assisted with sample collection and processing. RD collected samples from patients, provided laboratory space, and edited the manuscript. MT and KB designed the experiment and edited the manuscript. All authors contributed to the article and approved the submitted version.

## Conflict of Interest

The authors declare that the research was conducted in the absence of any commercial or financial relationships that could be construed as a potential conflict of interest.

## Supplementary Material

Figure S1 Box plots depicting alpha diversity comparisons between the groups in this study (healthy, control, CRSsNP or CRSwNP) according to **(A)** number of observed ASVs, **(B)** Shannon, or **(C)** Inverse Simpson metrics. Median values are indicated by the solid black line within each box, extending to the upper and lower quartile values. Outlier data points are indicated as closed black circles. Significant differences between groups are shown above each box with an asterisk (^*^).

Figure S2 Box plots depicting those ASVs which were identified as having significantly different relative sequence abundances between healthy (*n* = 20), control (*n* = 9), CRSsNP (*n* = 15), and CRSwNP (*n* = 16) groups. Kruskal-Wallis rank sum test generated overall *p*-values that indicated a significant difference existed at least once, and then pairwise comparisons were made between treatments using Dunn's test with “BH” *p*-value adjustment (precise *p*-values are in Table S2). Median values are indicated by the solid black line within each box, extending to the upper and lower quartile values. Outlier data points are indicated as closed black circles.

Table S1 Amplicon sequence variants (ASVs) assigned to Archaea that were detected in this study. Archaeal ASV sequences were used for placement in archaeal phylogenetic trees. ^*^Archaeal ASVs detected only in samples, ^**+**^Archaeal ASVs detected only in PCR controls, “__” indicates an ASV could not be confidently assigned at this taxonomic resolution.

Table S2 Evaluation of differences in microbial communities between groups at ASV level. Kruskal-Wallis rank sum test generated overall *p*-values that indicated a significant difference existed at least once across the groups, then pairwise comparisons were made between groups using Dunn's test with “BH” *p*-value correction for multiple pairwise comparisons. *p* < 0.05 were considered significant and are shown. __ = non-significant *p* > 0.05.
