# Supplementary figures and images for: A Novel Description of the Human Sinus Archaeome During Health and Chronic Rhinosinusitis
Source: Front Cell Infect Microbiol. 2020 Aug 6;10:398. doi: 10.3389/fcimb.2020.00398 (PMC7423975; doi:10.3389/fcimb.2020.00398)

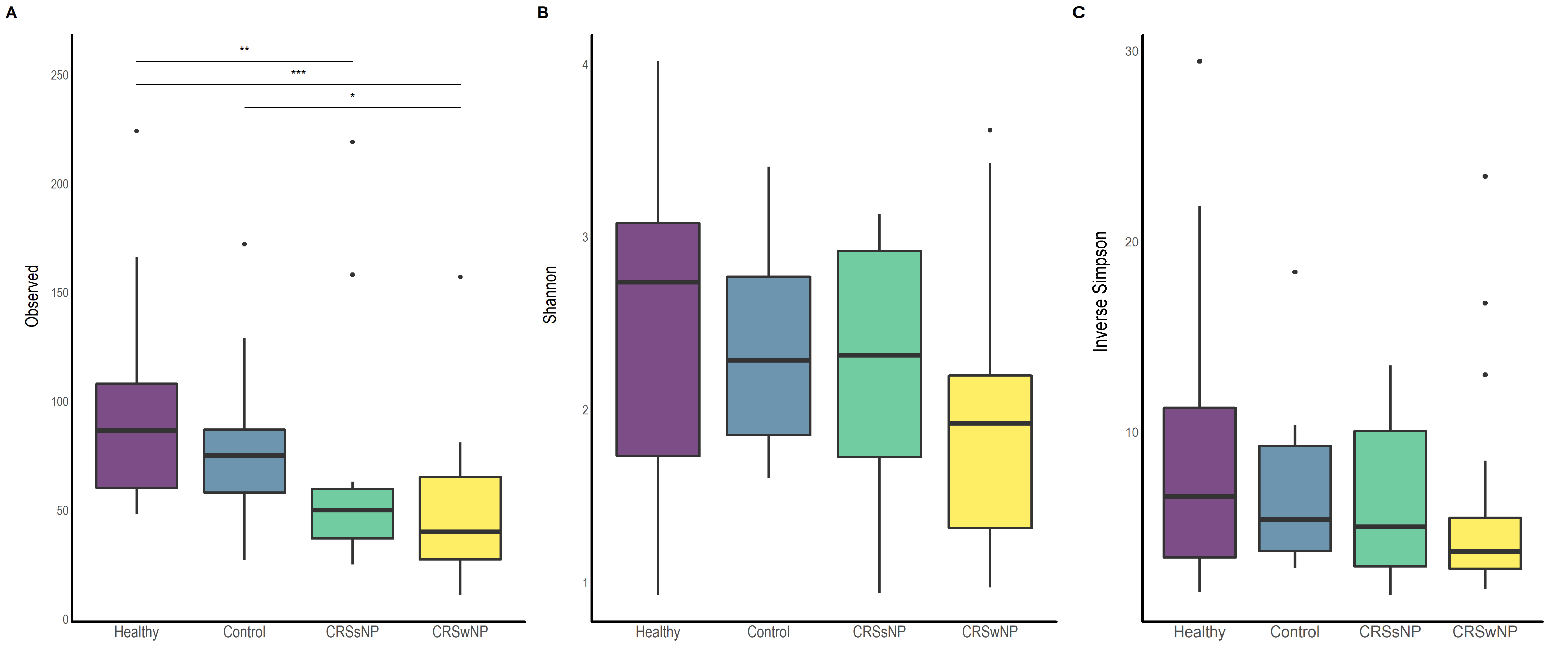

Supplement: Figure S1 — Box plots depicting alpha diversity comparisons between the groups in this study (healthy, control, CRSsNP or CRSwNP) according to (A) number of observed ASVs, (B) Shannon, or (C) Inverse Simpson metrics. Median values are indicated by the solid black line within each box, extending to the upper and lower quartile values. Outlier data points are indicated as closed black circles. Significant differences between groups are shown above each box with an asterisk (*). [file Image_1.PNG]

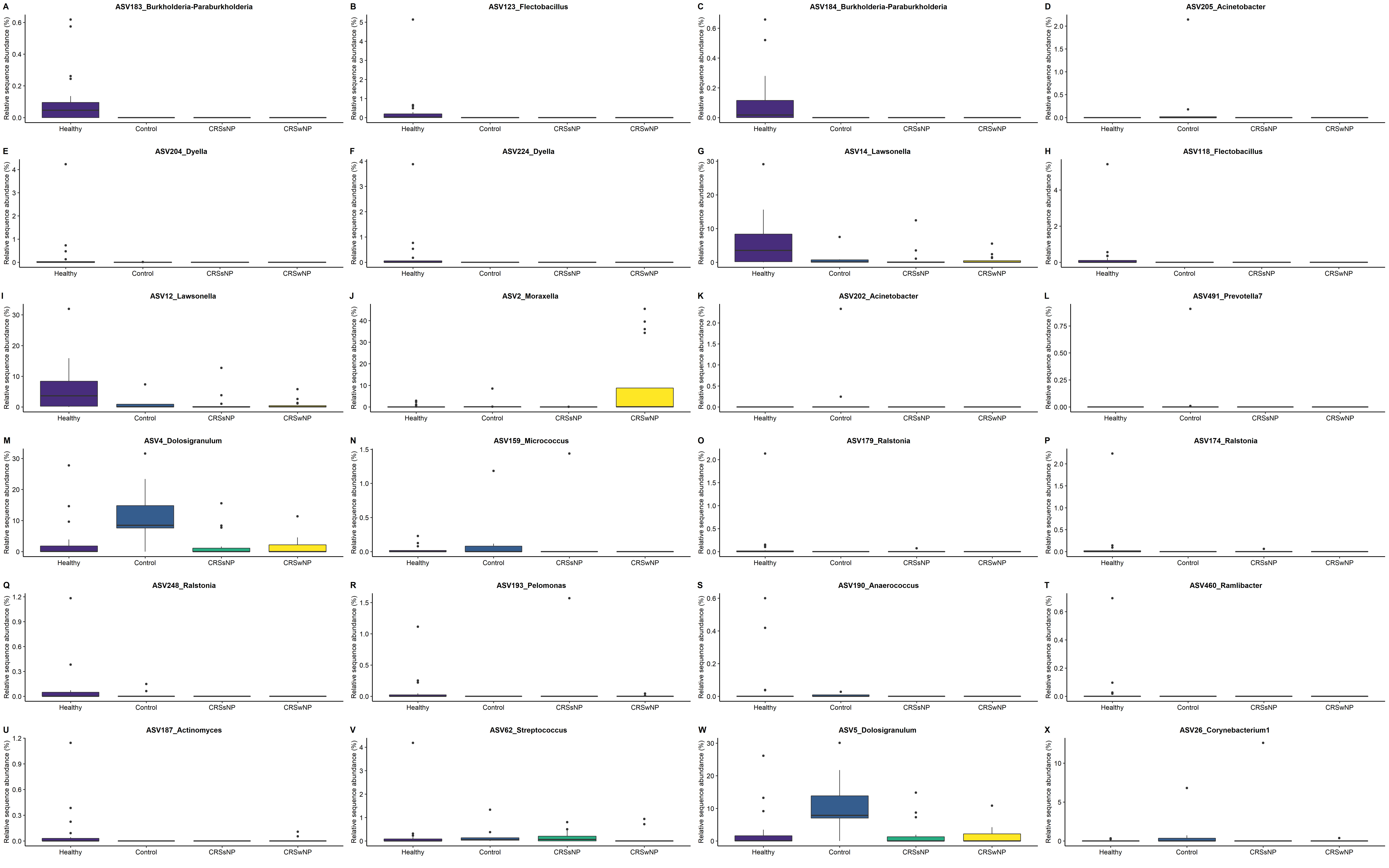

Supplement: Figure S2 — Box plots depicting those ASVs which were identified as having significantly different relative sequence abundances between healthy (n = 20), control (n = 9), CRSsNP (n = 15), and CRSwNP (n = 16) groups. Kruskal-Wallis rank sum test generated overall p-values that indicated a significant difference existed at least once, and then pairwise comparisons were made between treatments using Dunn's test with “BH” p-value adjustment (precise p-values are in Table S2). Median values are indicated by the solid black line within each box, extending to the upper and lower quartile values. Outlier data points are indicated as closed black circles. [file Image_2.JPEG]
