# Supplementary material for: A Novel Description of the Human Sinus Archaeome During Health and Chronic Rhinosinusitis
Source: Front Cell Infect Microbiol. 2020 Aug 6;10:398. doi: 10.3389/fcimb.2020.00398 (PMC7423975; doi:10.3389/fcimb.2020.00398)
Supplement: Table S1 — Amplicon sequence variants (ASVs) assigned to Archaea that were detected in this study. Archaeal ASV sequences were used for placement in archaeal phylogenetic trees. *Archaeal ASVs detected only in samples, +Archaeal ASVs detected only in PCR controls, “__” indicates an ASV could not be confidently assigned at this taxonomic resolution. [file Table_1.docx]

Table S1. Amplicon sequence variants (ASVs) assigned to Archaea that were detected in this study. Archaeal ASV sequences were used for placement in archaeal phylogenetic trees. **^*^**Archaeal ASVs detected only in samples, **^+^**Archaeal ASVs detected only in PCR controls, ‘__’ indicates an ASV could not be confidently assigned at this taxonomic resolution.

| ASV number | ASV sequence | Kingdom | Phylum | Class | Order | Family | Genus |
| --- | --- | --- | --- | --- | --- | --- | --- |
| Archaeal ASV1 | CCGGCAGTCCAAGTGATGGCCGCTATTATTGGGCCTAAAGCGTCCGTAGCCAGCCAGACAGGTCCGTCGGGAAATCCACGCGCTCAACGCGTGGGCGTCCGGCGGAAACCAGCTGGCTTGGGGCCGGAAGACCTGAGGGGTACGTCCGGGGTAGGAGTGAAATCCTGTAATCCTGGACGGACCACCGGTGGCGAAAGCGCCTCAGGAAGACGGACCCGACGGTGAGGGACGAAAGCTAGGGTCACGAACCGGATTAGATACCC | *Archaea* | *Euryarchaeota* | *Halobacteria* | *Halobacteriales* | *Halobacteriaceae* | *Halorussus* |
| Archaeal ASV2**^*^** | CCGGCAGCCCAAGTGATGGCCGCTGTTATTGGGCCTAAAGCGTCCGTAGCTGGCCGCACAAGTCCGTCGGGAAATCCGCCCGCTCAACGGGCGGCCGTCCGGCGGAACCTGTGTGGCTTGGGACCGGGAGGCCTGAGGGGTACGATCGGGGTAGGAGTGAAATCCTGTAATCCCGCTCGGACCGCCGATGGGGAAACCACCTCAGGAGAACGGATCCGACAGTGAGGGACGAAAGCCAGGGTCTCGAACCGGATTAGATACCC | *Archaea* | *Euryarchaeota* | *Halobacteria* | *Halobacteriales* | *Halobacteriaceae* | *Haloparvum* |
| Archaeal ASV3**^*^** | CCAGCACCCCGAGTGGTCGGGACGATTATTGGGCCTAAAGCATCCGTAGCCGGTTCTACAAGTCTTCCGTTAAATCCACCTGCTCAACAGTTGGGCTGCGGAGGATACTATAGAGCTAGGAGGCGGGAGAGGCAAGCGGTACTTAGTGGGTAGGGGTAAAATCCGTTGATCCATTGAAGACCACCAGTGGCGAAGGCGGCTTGCCAGAACGCACTCGACGGTGAGGGATGAAAGCTGGGGGAGCAAACCGGATTAGATACCCC | *Archaea* | *Thaumarchaeota* | *Soil_Crenarchaeotic_Group(SCG)* | *__* | *__* | *__* |
| Archaeal ASV4**^*^** | CCAGCACCCCGAGTGGTCGGGACGATTATTGGGCCTAAAGCATCCGTAGCCGGTTCTACAAGTCTTCCGTTAAATCCACCTGCTCAACAGTTGGGCTGCGGAGGATACTATAGAGCTAGGAGGCGGGAGAGGCAAGCGGTACTTAGTGGGTAGGGGTAAAATCCGTTGATCCATTGAAGACCACCAGTGGCGAAGGCGGCTTGCCAGAACGCACTCGACGGTGAGGGATGAAAGCTGGGGGAGCAAACCGGATTAGATACCCG | *Archaea* | *Thaumarchaeota* | *Soil_Crenarchaeotic_Group(SCG)* | *__* | *__* | *__* |
| Archaeal ASV5**^*^** | CCAGCACCCCGAGTGGTCGGGACGTTTATTGGGCCTAAAGCATCCGTAGCCGGTTCTACAAGTCTTCCGTTAAATCCACCTGCTTAACAGATGGGCTGCGGAAGATACTATAGAGCTAGGAGGCGGGAGAGGCAAGCGGTACTCGATGGGTAGGGGTAAAATCCGTTGATCCATTGAAGACCACCAGTGGCGAAGGCGGCTTGCCAGAACGCGCTCGACGGTGAGGGATGAAAGCTGGGGGAGCAAACCGGATTAGATACCCC | *Archaea* | *Thaumarchaeota* | *Soil_Crenarchaeotic_Group(SCG)* | *__* | *__* | *__* |
| Archaeal ASV6**^*^** | CCAGCACCCCGAGTGGTCGGGACGTTTATTGGGCCTAAAGCATCCGTAGCCGGTTCTACAAGTCTTCCGTTAAATCCACCTGCTTAACAGATGGGCTGCGGAAGATACTATAGAGCTAGGAGGCGGGAGAGGCAAGCGGTACTCGATGGGTAGGGGTAAAATCCGTTGATCCATTGAAGACCACCAGTGGCGAAGGCGGCTTGCCAGAACGCGCTCGACGGTGAGGGATGAAAGCTGGGGGAGCAAACCGGATTAGATACCCG | *Archaea* | *Thaumarchaeota* | *Soil_Crenarchaeotic_Group(SCG)* | *__* | *__* | *__* |
| Archaeal ASV7**^*^** | CCGGCAGCCCGAGTGATGGCCGATATTATTGGGCCTAAAGCGTCCGTAGCTGGCCGCGTAGGTCCGTCGGGAAATCTACTGGCTTAACCAGTAGGCGTCCGGCGGAAACCTCGTGGCTTGGGACCGGAAGACCTGAGGGGTACGTCTGGGGTAGGAGTGAAATCCCGTAATCCTGGACGGACCACCGGTGGCGAAAGCGCCTCAGGAAGACGGATCCGACAGTGAGGGACGAAAGCTAGGGTCTCGAACCGGATTAGATACCC | *Archaea* | *Euryarchaeota* | *Halobacteria* | *Halobacteriales* | *Halobacteriaceae* | *__* |
| Archaeal ASV8**^*^** | CCGGCAGCCCAAGTGATGGCCGCTGTTATTGGGCCTAAAGCGTCCGTAGCTGGCCGCACAAGTCCGTCGGGAAATCCGCCCGCTCAACGGGCGGCCGTCCGGCGGAACCTGTGCGGCTTGGGACCGGGAGGCCTGAGGGGTACGATCGGGGTAGGAGTGAAATCCTGTAATCCCGCTCGGACCGCCGATGGGGAAACCACCTCAGGAGAACGGATCCGACAGTGAGGGACGAAAGCCAGGGTCTCGAACCGGATTAGATACCC | *Archaea* | *Euryarchaeota* | *Halobacteria* | *Halobacteriales* | *Halobacteriaceae* | *Haloparvum* |
| Archaeal ASV9**^*^** | CCGGCAGCCCAAGTGATGGCCGCTGTTATTGGGCCTAAAGCGTCCGTAGCTGGCCGCACAAGTCCGTCGGGAAATCCGCCCGCTCAACGGGCGGCCGTCCGGCGGAACCTGTGTGGCCTGGGACCGGGAGGCCTGAGGGGTACGATCGGGGTAGGAGTGAAATCCTGTAATCCCGCTCGGACCGCCGATGGGGAAACCACCTCAGGAGAACGGATCCGACAGTGAGGGACGAAAGCCAGGGTCTCGAACCGGATTAGATACCC | *Archaea* | *Euryarchaeota* | *Halobacteria* | *Halobacteriales* | *Halobacteriaceae* | *Haloparvum* |
| Archaeal ASV10**^*^** | CCAGCACCCCGAGTGGTCGGGACGTTTATTGGGCCTAAAGCATCCGTAGCCGGTTCTACAAGTCTTCCGTTAAATCCACCTGCTTAACAGATGGACTGCGGAAGATACTATAGAGCTAGGAGGCGGGAGAGGCAAGCGGTACTCGATGGGTAGGGGTAAAATCCGTTGATCCATTGAAGACCACCAGTGGCGAAGGCGGCTTGCCAGAACGCGCTCGACGGTGAGGGATGAAAGCTGGGGGAGCAAACCGGATTAGATACCCG | *Archaea* | *Thaumarchaeota* | *Soil_Crenarchaeotic_Group(SCG)* | *__* | *__* | *__* |
| Archaeal ASV11**^*^** | CCAGCACCCCGAGTGGTCGGGACGTTTATTGGGCCTAAAGCATCCGTAGCCGGTTCTACAAGTCTTCCGTTAAATCCACCTGCTTAACAGATGGACTGCGGAAGATACTATAGAGCTAGGAGGCGGGAGAGGCAAGCGGTACTCGATGGGTAGGGGTAAAATCCGTTGATCCATTGAAGACCACCAGTGGCGAAGGCGGCTTGCCAGAACGCGCTCGACGGTGAGGGATGAAAGCTGGGGGAGCAAACCGGATTAGATACCCC | *Archaea* | *Thaumarchaeota* | *Soil_Crenarchaeotic_Group(SCG)* | *__* | *__* | *__* |
| Archaeal ASV12**^*^** | CCAGCACCCCGAGTGGTCGGGACGATTATTGGGCCTAAAGCATCCGTAGCCGGTTCTACAAGTCTTCCGTTAAATCCACCTGCTCAACAGTTGGGCTGCGGAGGATACTATAGAGCTAGGAGGCGGGAGAGGCAAGCGGTACTTAGTGGGTAGGGGTAAAATCCGTTGATCCATTGAAGACCACCAGTGGCGAAGGCGGCCTGCCAGAACGCACTCGACGGTGAGGGATGAAAGCTGGGGGAGCAAACCGGATTAGATACCCC | *Archaea* | *Thaumarchaeota* | *Soil_Crenarchaeotic_Group(SCG)* | *__* | *__* | *__* |
| Archaeal ASV13**^*^** | CCAGCACCCCGAGTGGTCGGGACGATTATTGGGCCTAAAGCATCCGTAGCCGGTTCTACAAGTCTTCCGTTAAATCCACCTGCTCAACAGTTGGGCTGCGGAGGATACTATAGAGCTAGGAGGCGGGAGAGGCAAGCGGTACTTAGTGGGTAGGGGTAAAATCCGTTGATCCATTGAAGACCACCAGTGGCGAAGGCGGCCTGCCAGAACGCACTCGACGGTGAGGGATGAAAGCTGGGGGAGCAAACCGGATTAGATACCCG | *Archaea* | *Thaumarchaeota* | *Soil_Crenarchaeotic_Group(SCG)* | *__* | *__* | *__* |
| Archaeal ASV14**^*^** | CCGGCAGCTCTAGTGGTAGCAGTTTTTATTGGGCCTAAAGCGTCCGTAGCCGGTTTAATAAGTCTCTGGTGAAATCCTGCAGCTTAACTGTGGGAATTGCTGGAGATACTATTAGACTTGAGATCGGGAGAGGTTAGAGGTACTCCCAGGGTAGAGGTGAAATTCTGTAATCCTGGGAGGACCGCCTGTTGCGAAGGCGTCTGACTGGAACGATTCTGACGGTGAGGGACGAAAGCTAGGGGCGCGAACCGGATTAGATACCC | *Archaea* | *Euryarchaeota* | *Methanobacteria* | *Methanobacteriales* | *Methanobacteriaceae* | *Methanobrevibacter* |
| Archaeal ASV15**^*^** | CCGGCAGCTCAAGTGGTGGCCGTTTTTATTGGGCCTAAAGCGTTCGTAGCCGGCCTGATAAGTCTCTGGTGAAATCCCGCAGCTTAACTGTGGGAATTGCTGGAGATACTATCAGGCTTGAGGTCGGGAGAGGTTAGAGGTACTCCCAGGGTAGGGGTGAAATCCTATAATCCTGGGAGGACCACCTGTGGCGAAGGCGTCTAACTGGAACGAACCTGACGGTGAGTAACGAAAGCCAGGGGCGCGAACCGGATTAGATACCC | *Archaea* | *Euryarchaeota* | *Methanobacteria* | *Methanobacteriales* | *Methanobacteriaceae* | *Methanobacterium* |
| Archaeal ASV16**^*^** | CCGGCGGCTCGAGTGGTAACCGTTATTATTGGGTCTAAAGGGTCTGTAGCCGGCCGGATAAGTCTCTTGAGAAATCTGGCAGCTTAACTGTCAGGCTTTCAGGAGATACTGTCTGGCTCGAGGCCGGGAGAGGTGAGAGGTACTTCAGGGGTAGGGGTGAAATCTTGTAATCCTTGAAGGACCACCAGTGGCGAAGGCGTCTCACCAGAACGGACCTGACGGCAAGGGACGAAAGCTAGGGGCACGAACCGGATTAGATACCC | *Archaea* | *Euryarchaeota* | *Methanomicrobia* | *Methanosarcinales* | *Methanosaetaceae* | *Methanosaeta* |
| Archaeal ASV17**^+^** | CCCGCAGCTCAAGTGGTGGCCACTATTATTGAGCCTAAAGCGTCCGTAGCCGGTCTTGTAAATCTCTGGGTAAATCCCGCCGCTCAACGGTGGGAAGACTGGAGAGACTGCAAGACTAGGGATCGGGTGAGGTAAGAGGTACTCTTGGGGTAGGGGTAAAATCCTGTAATCCTGAGGGGACGACCGGTGGCGAAGGCGTCTTACTAGAACGACTCCGACGGTGAGGGACGAAGGCTAGGGGAGCAAACCGGATTAGATACCCC | *Archaea* | *Euryarchaeota* | *Thermoplasmata* | *Thermoplasmatales* | *BSLdp215* | *__* |
| Archaeal ASV18**^+^** | CCAGCACCCCGAGTGGTCGGGACGATTATTGGGCCTAAAGCATCCGTAGCTGGTATAACAAGTCCTCCGTTAAATCCACCTGCTTAACAGGTGGGCTGCGGAGGATACTGTTACACTAGGAGGCGGGAGAGGCGAGGGGTATTCCACGGGTAGGGGTAAAATCCTCTGATCCGTGGAGGACTACCAGTGGCGAAGGCGCCTCGCTAGAACGCGCTCGACGGTGAGGGATGAAAGCTGGGGGAGCAAACCGGATTAGATACCCG | *Archaea* | *Thaumarchaeota* | *__* | *__* | *__* | *__* |
| Archaeal ASV19**^+^** | CCGGCAGTCCAAGTGATGGCCGCTATTATTGGGCCTAAAGCGTCCGTAGCCAGCCAGGCAGGTCCGTCGGGAAATCCACGCGCTCAACGCGTGGGCGTCCGGCGGAAACCAGCTGGCTTGGGGCCGGAAGACCTGAGGGGTACGTCCGGGGTAGGAGTGAAATCCTGTAATCCTGGACGGACCACCGGTGGCGAAAGCGTCTGACTAGAACGGCTCTGACGGTGAGGAACGAAGGCTAGGGGAGCAAACCGGATTAGATACCC | *Archaea* | *Euryarchaeota* | *Halobacteria* | *Halobacteriales* | *Halobacteriaceae* | *Halorussus* |
