# Supplementary material for: A Novel Description of the Human Sinus Archaeome During Health and Chronic Rhinosinusitis
Source: Front Cell Infect Microbiol. 2020 Aug 6;10:398. doi: 10.3389/fcimb.2020.00398 (PMC7423975; doi:10.3389/fcimb.2020.00398)
Supplement: Table S2 — Evaluation of differences in microbial communities between groups at ASV level. Kruskal-Wallis rank sum test generated overall p-values that indicated a significant difference existed at least once across the groups, then pairwise comparisons were made between groups using Dunn's test with “BH” p-value correction for multiple pairwise comparisons. p < 0.05 were considered significant and are shown. __ = non-significant p > 0.05. [file Table_2.docx]

Table S2. Evaluation of differences in microbial communities between groups at ASV level. Kruskal-Wallis rank sum test generated overall *p*-values that indicated a significant difference existed at least once across the groups, then pairwise comparisons were made between groups using Dunn’s test with “BH” *p*-value correction for multiple pairwise comparisons. *p*-values <0.05 were considered significant and are shown. __ = non-significant *p*-value > 0.05.

| ASV | Phylum | Genus | Control - CRSsNP | Control - CRSwNP | CRSsNP - CRSwNP | Control - Healthy | CRSsNP - Healthy | CRSwNP - Healthy |
| --- | --- | --- | --- | --- | --- | --- | --- | --- |
| ASV183 | *Proteobacteria* | *Burkholderia-Paraburkholderia* | __ | __ | __ | 0.000 | 0.000 | 0.000 |
| ASV123 | *Bacteroidetes* | *Flectobacillus* | __ | __ | __ | 0.000 | 0.000 | 0.000 |
| ASV184 | *Proteobacteria* | *Burkholderia-Paraburkholderia* | __ | __ | __ | 0.001 | 0.000 | 0.000 |
| ASV205 | *Proteobacteria* | *Acinetobacter* | 0.000 | 0.000 | __ | 0.000 | __ | __ |
| ASV204 | *Proteobacteria* | *Dyella* | __ | __ | __ | 0.032 | 0.001 | 0.002 |
| ASV224 | *Proteobacteria* | *Dyella* | __ | __ | __ | 0.007 | 0.002 | 0.004 |
| ASV14 | *Actinobacteria* | *Lawsonella* | __ | __ | __ | 0.016 | 0.002 | 0.004 |
| ASV118 | *Bacteroidetes* | *Flectobacillus* | __ | __ | __ | 0.014 | 0.006 | 0.009 |
| ASV12 | *Actinobacteria* | *Lawsonella* | __ | __ | __ | 0.039 | 0.006 | 0.011 |
| ASV2 | *Proteobacteria* | *Moraxella* | 0.020 | __ | 0.005 | __ | __ | 0.045 |
| ASV202 | *Proteobacteria* | *Acinetobacter* | 0.004 | 0.005 | __ | 0.007 | __ | __ |
| ASV491 | *Bacteroidetes* | *Prevotella_7* | 0.004 | 0.005 | __ | 0.007 | __ | __ |
| ASV4 | *Firmicutes* | *Dolosigranulum* | 0.007 | 0.010 | __ | 0.007 | __ | __ |
| ASV159 | *Actinobacteria* | *Micrococcus* | 0.034 | 0.016 | __ | __ | __ | 0.030 |
| ASV179 | *Proteobacteria* | *Ralstonia* | __ | __ | __ | 0.031 | 0.032 | 0.017 |
| ASV174 | *Proteobacteria* | *Ralstonia* | __ | __ | __ | 0.032 | 0.035 | 0.018 |
| ASV248 | *Proteobacteria* | *Ralstonia* | __ | __ | __ | __ | 0.016 | 0.029 |
| ASV193 | *Proteobacteria* | *Pelomonas* | __ | __ | __ | 0.035 | 0.025 | 0.033 |
| ASV190 | *Firmicutes* | *Anaerococcus* | 0.034 | 0.064 | __ | __ | 0.044 | 0.054 |
| ASV460 | *Proteobacteria* | *Ramlibacter* | __ | __ | __ | 0.048 | 0.030 | 0.054 |
| ASV187 | *Actinobacteria* | *Actinomyces* | __ | __ | __ | 0.043 | 0.031 | __ |
| ASV62 | *Firmicutes* | *Streptococcus* | __ | 0.035 | __ | __ | __ | __ |
| ASV5 | *Firmicutes* | *Dolosigranulum* | 0.023 | 0.032 | __ | 0.016 | __ | __ |
| ASV26 | *Actinobacteria* | *Corynebacterium_1* | 0.020 | 0.031 | __ | 0.031 | __ | __ |
